# Secret Commissions

**Published:** 1899-09-09

**Authors:** 


### Secret Commissions.

Sir Edward Fry has descended upon the medical profession in a "bolt from the blue." In the very middle of the silly season, when the leading London doctors are scattered to the four points of the compass, he succeeds in obtaining the insertion in the *Times* of a letter more than a column in length, in which he maintains that "a noble profession," as he admits it to be, "is not untainted in its lower walks by the vice of secret commissions"; and in support of this contention he seems to have no other evidence than the assertion of a "pharmaceutical chemist" to the Committee of the London Chamber of Commerce that "secret commissions are given by chemists to medical men on their prescriptions supplied to patients, in some cases amounting to from 25 to 50 per cent. on the amount charged," and the kindred assertion of "an optician, jeweller, and silversmith of fifty years' experience" that it is "an open secret that hospital doctors receive commissions from makers of surgical instruments." Considering that hospitals do not obtain their instruments from an "optician, jeweller, and silversmith," and that "hospital doctors" are not usually described as being in "the lower walks of the profession," it does not appear to us that Sir Edward's second witness renders any material support to his contention. With regard to the first, if his statement had any foundation in fact at all, it would probably be that a general practitioner, who had been accustomed to dispense his own medicines, entered into an arrangement with a neighbouring chemist to cease from doing so, and to send his prescriptions to the chemist's shop, on consideration of receiving a share of the charges made for them. It might be worth the while of a chemist to enter into such an agreement, not only for the sake of the portion of profit which would still remain to him, but also for the sake of the additional general business which the patients might be expected to bring. From a medical point of view, the arrangement could not be considered either laudable or desirable; but it need not even be "secret," and it certainly need not be corrupt. It would not be to the interest of the doctor that his patients should be called upon to pay more than ordinary prices for their medicines, and it would be to his interest to secure that the drugs employed were of good quality and well dispensed. The evil of "secret commissions" begins when they sacrifice the consumer, and cause him either to pay an unfair price or to receive an inferior article. As for the story about instruments, it is utterly absurd upon the face of it. A hospital doctor (Sir E. Fry presumably means surgeon) holds his appointment for the sake of the reputation which, if its duties are well fulfilled, it is calculated to bring; and the quality and trustworthiness of the instruments supplied to the institution are important factors in his own success. To suppose that he would tolerate inferior instruments for the sake of earning a small commission is too absurd for ordinary credulity.

Then come some stories about commissions to doctors from undertakers. There is, or was, a large firm of undertakers in London, and there is, or was, a large firm of the same name, doing a considerable business as jobmasters, and rather laying themselves out for horsing doctors' carriages. Possibly the two firms were practically the same; and, at all events, we believe members of the same family were concerned in the management of both. We have more than once heard it said as a joke that the jobmasters horsed Dr. So-and-so's carriage, and that no doubt they did it on easy terms for the sake of the funerals which in due time followed in his wake. We never regarded the imputation seriously; and the only instance we have ever known in which a commission was asked for by a doctor was in the case of a foreign ophthalmic surgeon, who at one time enjoyed a considerable practice in London. In a profession of twenty-five thousand persons, brought up under various conditions and subjected to varying temptations, it can hardly be expected that all hands should be clean; but we do not hesitate to say that Sir Edward Fry's zeal in a good cause has, on this occasion, led him into errors arising from undue haste or undue credulity. It is with no disrespect to his judicial ability that we suggest to him more care in accepting and sifting what appears to pass with him for evidence, and more pains in ascertaining the relation which the British Medical Association, or its Council, really bears to professional opinion or professional conduct. The only bodies in England by which conduct short of crime, or of the technical definition of "infamy," can be controlled are the Royal Colleges of Physicians and Surgeons. The Medical Council and the Society of Apothecaries are alike powerless to deal with minor evils.

